# Shear Stress Modulation of Smooth Muscle Cell Marker Genes in 2-D and 3-D Depends on Mechanotransduction by Heparan Sulfate Proteoglycans and ERK1/2

**DOI:** 10.1371/journal.pone.0012196

**Published:** 2010-08-16

**Authors:** Zhong-Dong Shi, Giya Abraham, John M. Tarbell

**Affiliations:** Department of Biomedical Engineering, The City College of New York, The City University of New York (CUNY), New York, New York, United States of America; Ohio State University, United States of America

## Abstract

**Background:**

During vascular injury, vascular smooth muscle cells (SMCs) and fibroblasts/myofibroblasts (FBs/MFBs) are exposed to altered luminal blood flow or transmural interstitial flow. We investigate the effects of these two types of fluid flows on the phenotypes of SMCs and MFBs and the underlying mechanotransduction mechanisms.

**Methodology/Principal Findings:**

Exposure to 8 dyn/cm^2^ laminar flow shear stress (2-dimensional, 2-D) for 15 h significantly reduced expression of α-smooth muscle actin (α-SMA), smooth muscle protein 22 (SM22), SM myosin heavy chain (SM-MHC), smoothelin, and calponin. Cells suspended in collagen gels were exposed to interstitial flow (1 cmH_2_O, ∼0.05 dyn/cm^2^, 3-D), and after 6 h of exposure, expression of SM-MHC, smoothelin, and calponin were significantly reduced, while expression of α-SMA and SM22 were markedly enhanced. PD98059 (an ERK1/2 inhibitor) and heparinase III (an enzyme to cleave heparan sulfate) significantly blocked the effects of laminar flow on gene expression, and also reversed the effects of interstitial flow on SM-MHC, smoothelin, and calponin, but enhanced interstitial flow-induced expression of α-SMA and SM22. SMCs and MFBs have similar responses to fluid flow. Silencing ERK1/2 completely blocked the effects of both laminar flow and interstitial flow on SMC marker gene expression. Western blotting showed that both types of flows induced ERK1/2 activation that was inhibited by disruption of heparan sulfate proteoglycans (HSPGs).

**Conclusions/Significance:**

The results suggest that HSPG-mediated ERK1/2 activation is an important mechanotransduction pathway modulating SMC marker gene expression when SMCs and MFBs are exposed to flow. Fluid flow may be involved in vascular remodeling and lesion formation by affecting phenotypes of vascular wall cells. This study has implications in understanding the flow-related mechanobiology in vascular lesion formation, tumor cell invasion, and stem cell differentiation.

## Introduction

The major functions of vascular smooth muscle cells (SMCs) are to maintain and regulate blood vessel tone, blood pressure, and blood flow distribution. SMCs retain remarkable plasticity and can undergo phenotypic modulation between contractile state and synthetic state in response to alterations in local environmental cues [1], [2]. The phenotype of SMC is a continuum and thus the phenotype state refers to relative position along the continuum, indicating cell marker expression and functions that are associated with either a contractile or a synthetic state [3]. In response to injury, medial SMCs can dramatically increase proliferation, motility, and secretion capacity, and play critical roles in vascular repair and remodeling [1], [2], [4]. However, if the responses are excessive, SMCs may also contribute to vascular lesion formation by migrating from the media into the intima under abnormal environmental conditions [1], [5]. Besides SMCs, adventitial fibroblasts (FBs) and their activated counterpart myofibroblasts (MFBs) are also involved in vascular lesion formation [6], [7].

Vascular SMCs and FBs/MFBs normally reside in a 3-dimensional (3-D) environment composed of extracellular matrix (ECM) components mainly collagen I and III. Most in vitro studies have investigated responses of SMCs to chemical or mechanical stimuli by culturing them on 2-D substrates. However, it has been shown that 3-D culture systems are a better representation of the in vivo environment than conventional 2-D systems [2], [3]. In a 3-D collagen gel, SMCs are less proliferative and more quiescent compared with SMCs cultured in 2-D on a collagen matrix [8], [9].

The contractile SMCs in the media are exposed to a physiological interstitial flow driven by the transmural pressure differential [10], [11]. However, during vascular injury, SMCs may be exposed to elevated interstitial flow after damage to the vascular endothelium [5], and the superficial layer of SMCs may even be directly exposed to luminal blood flow where the intima is denuded. Modeling studies have shown that transmural interstitial flow passes through the oriented SMC layers to the adventitia and imposes shear stresses on SMCs and FBs that are of the order of 0.1 dyn/cm^2^, and could be lower or higher depending on the location of the cells in the vessel wall [5], [10], [11]. After intima damage, luminal blood flow imposes shear stress on the first layer of SMCs and this shear stress may be somewhat lower than that on endothelial cells (ECs) due to the local structure of the injury. In the early stages of injury, shear stresses (luminal blood flow and transmural interstitial flow) on SMCs are elevated, and have been hypothesized to contribute to neointima formation [5], [12]–[14]. During vascular repair or vascular lesion formation (takes hours to days or even weeks), shear stresses on SMCs are decreased. It has been shown that 2-D shear stress (∼10 dyn/cm^2^) can reduce expression of SMC marker genes [15], [16] and promote SMC proliferation [15], [17]. In addition, SMC and MFB have different migratory responses to laminar flow (2-D) and interstitial flow (3-D) [5], [13], [18].

To date, no studies have shown whether interstitial flow affects SMC and MFB phenotype in 3-D, and the mechanisms by which SMCs and MFBs sense fluid flow shear stress and modulate their phenotypes remain unclear. Given that switching SMC from contractile to synthetic phenotype will increase cell proliferation and motility, we therefore postulate that there can be some shared mechanisms between cell phenotypic switching and migration. We have already shown that ERK1/2 signaling plays a key role in interstitial flow-induced SMC motility [19]. In this study, we investigated how laminar flow and interstitial flow affect the expression of SMC marker genes and the potential role of ERK1/2. In addition, it has been suggested that cell surface glycocalyx component heparan sulfate proteoglycans (HSPGs) are shear stress sensors for endothelial cells and SMCs in 2-D [20]–[23]. There has been no study to show whether or not the glycocalyx functions as an interstitial flow sensor in 3-D. Therefore we also examined the role of HSPGs in mechanotransduction in both 2-D and 3-D.

## Results

### Laminar flow reduces SMC marker gene expression in SMCs and MFBs in 2-D

**Figure 1:**
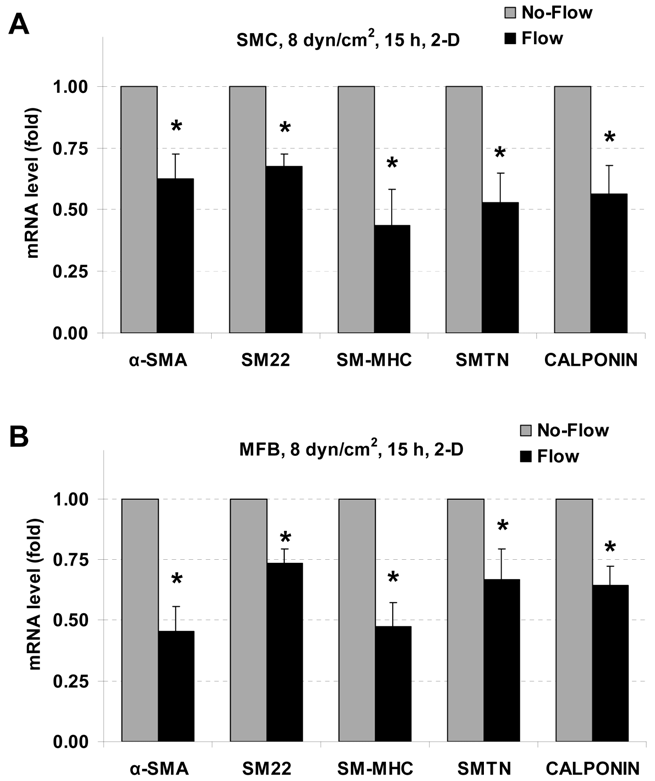
Effects of laminar flow on SMC marker gene expression in 2-D. Laminar flow downregulates SMC marker gene expression in both SMCs and MFBs in 2-D. SMCs (A) and MFBs (B) were exposed to 8 dyn/cm^2^ laminar shear stress for 15 h. Expression of SMC marker genes (α-SMA, SM22, SM-MHC, smoothelin (SMTN), and calponin) were analyzed by RT-qPCR. The gene expression was normalized to its companion No-Flow control case. All the data are presented as mean ± SEM. * P<0.05 vs corresponding No-Flow control; n = 4–6.

To investigate the effect of laminar shear stress (2-D) on SMC marker expression in SMCs and MFBs, cells were cultured in inserts (6-well format with 0.4 µm pore size; 1.5×10^5^ cells/filter) for 24 h. Cells were then exposed to 8 dyn/cm^2^ (average) shear stress applied by a rotating disk shear rod for 15 h. The mRNA levels were measured by RT-qPCR, and are shown in Figure 1. Exposure to 8 dyn/cm^2^ of laminar shear stress significantly reduced α-smooth muscle actin (α-SMA), smooth muscle protein 22 (SM22 also called as transgelin), smooth muscle myosin heavy chain (SM-MHC), smoothelin (SMTN), and calponin gene expression in both SMCs (Figure 1A) and MFBs (Figure 1B).

### Interstitial flow attenuates SM-MHC, SMTN, and calponin expression but enhances α-SMA and SM22 expression in 3-D

**Figure 2:**
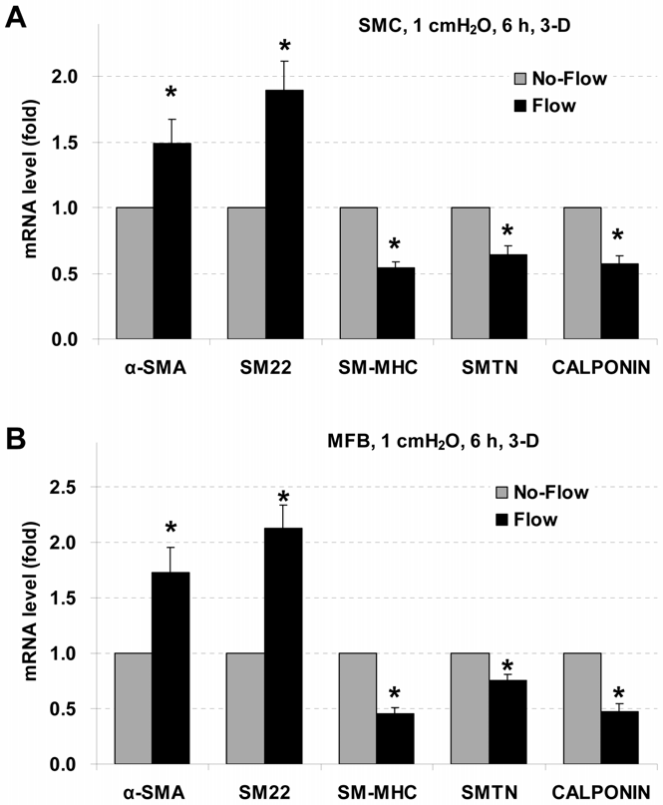
Effects of interstitial flow on SMC marker gene expression in 3-D. Interstitial flow attenuates expression of SM-MHC, smoothelin, and calponin, but promotes expression of α-SMA and SM22 in both SMCs and MFBs in 3-D. SMCs (A) and MFBs (B) in collagen I gels were exposed to interstitial fluid flow driven by 1 cmH_2_O pressure differential (∼0.05 dyn/cm^2^ shear stress [5]) for 6 h. Gene expression was analyzed by RT-qPCR and normalized to its own No-Flow control case. All the data are presented as mean ± SEM. * P<0.05 vs corresponding No-Flow control; n = 4–6.

To evaluate the influence of interstitial flow on SMC marker expression, SMCs and MFBs were suspended in collagen I gels and plated in cell culture inserts (6-well format with 8 µm pore size; 1 ml of 4 mg/ml collagen gel; cell density: 2.5×10^5^ cells/ml). After spreading in gels for 24 h, cells were then subjected to interstitial flow driven by 1 cmH_2_O pressure differential (∼0.05 dyn/cm^2^) for 6 h. The expression of SMC markers was assessed by RT-qPCR and shown in Figure 2. Just like laminar flow in 2-D, interstitial flow in 3-D also significantly reduced SMC marker SM-MHC, smoothelin, and calponin gene expression in both SMCs and MFBs (Figure 2). However, unlike laminar flow, interstitial flow markedly promoted α-SMA and SM22 expression in both cell types.

### PD98059 and heparinase III block laminar flow-induced reduction in SMC marker expression in 2-D

**Figure 3:**
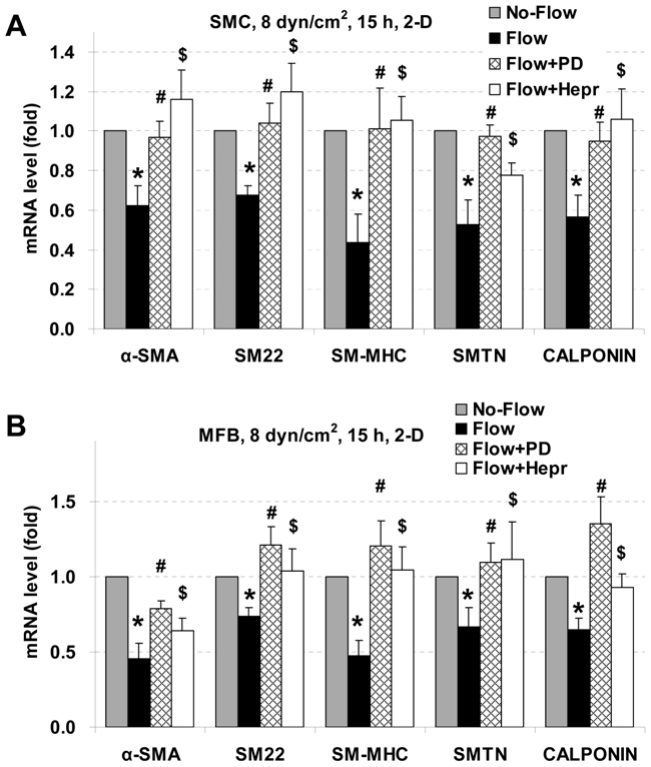
Effects of PD98059 and heparinase on SMC marker gene expression under 2-D laminar flow. PD98059 and heparinase reverse laminar flow-induced reductions in expression of SMC marker genes in 2-D. SMCs (A) and MFBs (B) were pretreated with PD98059 (PD) or heparinase III (Hepr) for 3 h, and then exposed to 8 dyn/cm^2^ laminar shear stress for 15 h. Gene expression was analyzed by RT-qPCR and normalized to its own Flow without PD or Hepr treated case. All the data are presented as mean ± SEM. * P<0.05 vs corresponding No-Flow control; #P<0.05 vs corresponding Flow case; $ P<0.05 vs corresponding Flow case; n = 4–5.

To investigate the underlying mechanisms by which laminar flow attenuated SMC marker expression, PD98059 was used to inhibit ERK1/2 activation and heparinase III was used to cleave cell surface heparan sulfate glycosaminoglycans (HS GAGs) from heparan sulfate proteoglycans (HSPGs). Cells were pre-incubated with 10 µM of PD98059 or 6.7 IU/L of heparinase for 3 h followed by exposure to laminar flow for 15 h. Flow medium contained 10 µM of PD98059 for ERK inhibition experiments, and flow medium contained 1.0 IU/L heparinase for HSPG cleavage experiments. RT-qPCR results are shown in Figure 3. After treatment with PD98059 or heparinase, reduction in gene expression in SMCs and MFBs caused by laminar flow were significantly blocked in both cell types. These data suggest that ERK1/2 MAPK and cell surface HSPGs played important roles in laminar flow-induced decreases in SMC marker expression.

### PD98059 and heparinase block interstitial flow-induced reduction in SM-MHC, SMTN, and calponin expression, but enhance α-SMA or SM22 expression in 3-D

**Figure 4:**
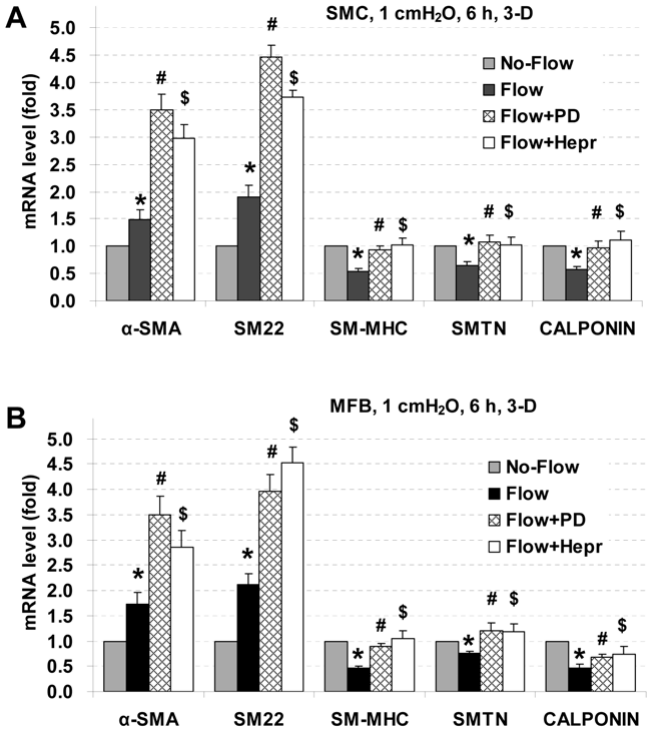
Effects of PD98059 and heparinase on SMC marker gene expression under 3-D interstitial flow. PD98059 and heparinase reverse interstitial flow-induced reductions in SM-MHC, smoothelin, and calponin expression, but further enhance α-SMA and SM22 expression in 3-D. SMCs (A) and MFBs (B) in collagen gels were pretreated with PD98059 (PD) or heparinase III (Hepr) for 3 h, and then exposed to interstitial flow (1 cmH_2_O) for 6 h. Gene expression was analyzed by RT-qPCR and normalized to its own Flow without PD or Hepr treated case. All the data are presented as mean ± SEM. * P<0.05 vs corresponding No-Flow control; #P<0.05 vs corresponding Flow case; $ P<0.05 vs corresponding Flow case; n = 4–5.

To examine whether the effects of interstitial flow on SMC marker gene expression were also related to ERK1/2 and HSPGs, cells were pre-incubated with 10 µM of PD98059 or 6.7 IU/L of heparinase for 3 h followed by exposure to interstitial flow for 6 h. Flow medium contained 10 µM of PD98059 for ERK inhibition experiments, and flow medium contained 1.0 IU/L heparinase for HSPG cleavage experiments. As shown in Figure 4, inhibition of ERK1/2 or cleavage of HSPGs eliminated interstitial flow-induced decreases in SM-MHC, smoothelin, and calponin expression in both SMCs and MFBs. However, either inhibition of ERK1/2 or removal of HSPGs did not attenuate interstitial flow-induced α-SMA and SM22 expression. Instead, expression of α-SMA and SM22 was further enhanced. These data suggest that ERK1/2 and HSPGs were involved in interstitial flow-mediated SMC marker expression.

### Knocking down ERK1/2 attenuates effects of both laminar flow and interstitial flow on SMC marker expression

**Figure 5:**
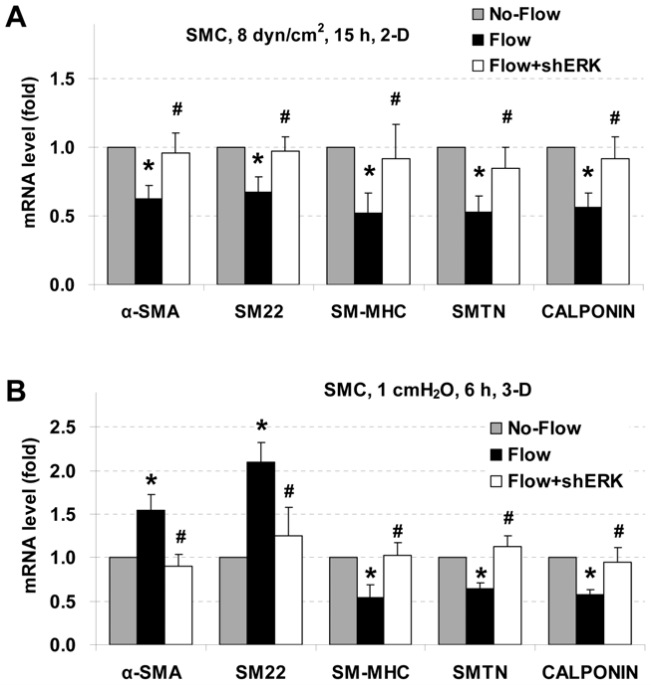
ERK1/2 knockdown reverses effects of both laminar flow and interstitial flow on SMC marker expression. The cells were transfected with ERK1/2 shRNAs or shRNA vectors and then were exposed to laminar flow (A) and interstitial flow (B). All the data were presented as mean ± SEM. * P<0.05 vs corresponding No-Flow vector control; #P<0.05 vs corresponding vector Flow case; n = 4.

Since PD98059 may have cellular effects other than inhibition of ERK1/2 [24], shRNA specific to ERK1/2 was used. Due to the high similarity in SMC marker gene expression between SMCs and MFBs when they were exposed to either laminar flow or interstitial flow, ERK1/2 gene knockdown was only performed in SMCs. Knockdown of ERK1/2 significantly reversed laminar flow-induced down-regulation of SMC marker gene expression in 2-D (Figure 5A). Silencing of ERK1/2 also significantly inhibited interstitial flow effects on SMC marker expression in 3-D: reduced expression of SM-MHC, smoothelin, and calponin were significantly reversed with ERK1/2 knockdown; while increased expression of both α-SMA and SM22 were markedly inhibited (Figure 5B). These results suggest that regulation of SMC marker gene expression by fluid flow is dependent on the ERK1/2 signaling pathway.

### Heparan sulfate proteoglycans are mechanosensors for fluid flow-induced and ERK1/2-mediated cell phenotypic switching

**Figure 6:**
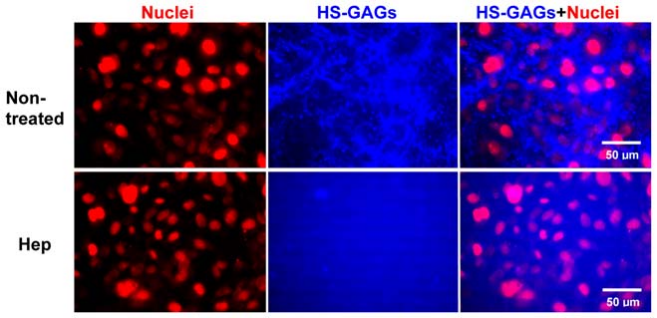
Cleavage of heparan sulfate glycosaminglycans (HS-GAGs) by heparinase. SMCs were grown on the plate for 2 days, and then incubated with 6.7 IU/L heparinase III (Hep) for 1 h followed by immunostaining for HS-GAGs. The surfaces of SMCs present abundant HS-GAGs (blue), which was successfully cleaved by heparinase III. Cell nuclei were stained by propidium iodide shown in red.

**Figure 7:**
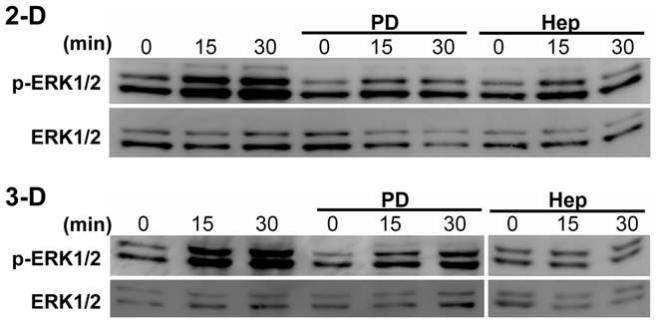
PD98059 and heparinase suppress both laminar flow and interstitial flow-induced ERK1/2 activation. Cells in 2-D or 3-D were pretreated with ERK1/2 inhibitor PD98059 (PD) or heparinase III (Hep) and then exposed to laminar flow or interstitial flow for 0 to 30 min. Cells were lysed and proteins were extracted for Western blotting. Gel panels were representative Western blots from three independent experiments, where similar results were found.

The efficiency of heparinase III in cleaving cell surface HSPG GAGs was evaluated by immunostaining. Figure 6 shows that SMC surfaces contain abundant HSPGs and that using heparinase III can substantially cleave cell surface HS GAGs. Removal of HSPGs reversed most of the flow effects, suggesting that HSPGs might be involved in ERK1/2 activation. To examine whether the fluid flow affected ERK1/2 activation and the role of cell surface HSPGs, Western blotting was used to determine ERK1/2 phosphorylation. The results are shown in Figure 7. Both laminar flow (2-D) and interstitial flow (3-D) dramatically induced ERK1/2 phosphorylation, and PD98059 significantly inhibited fluid flow-induced ERK1/2 activation. After removal of HSPGs, however, activation of ERK1/2 induced by fluid flow was significantly attenuated as well. These results suggest that ERK1/2 activation was mediated by cell surface HSPGs. Although PD98059 and heparinase had different effects on the expression of some SMC marker genes in 3-D (Figure 4) compared with 2-D (Figure 3), knockdown of ERK1/2 abolished flow effects in both 2-D and 3-D (Figure 5). In addition, both PD98059 and removal of HS-GAGs suppressed flow induced ERK1/2 activation in 2-D and 3-D (Figure 7), suggesting that besides displaying some other cellular effects, HSPGs are essential in mediating flow-induced ERK1/2 activation. Fluid flow slightly induced ERK1/2 activation after treatment with PD98059 and heparinase, but activation of ERK1/2 was significantly lower than the corresponding time points in non-treated cases (Figure 7). Therefore, we conclude that the effects of laminar flow and interstitial flow on expression of SMC marker genes depend on ERK1/2 activation via a mechanotransduction mechanism mediated by HSPGs.

## Discussion

It has been widely recognized that laminar flow shear stress has a great impact on EC biology [25]. Many other types of cells including SMCs, FBs, bone cells, stem cells and tumor cells in the tissue interstitium are exposed to a very low interstitial fluid flow [26]. Interstitial flow can modulate many cellular processes in a 3-D environment including proliferation, apoptosis, differentiation, and migration [5], [8], [27], [28]. For example, interstitial flow can induce cytokine release, vascular and tumor cell migration, capillary morphogenesis, and stem cell differentiation [5], [8], [19], [29]–[31]. Interstitial flow therefore plays important roles in tissue physiology and pathology.

In the present study, we demonstrated that laminar flow and interstitial flow significantly affect expression of SMC marker genes (α-SMA, SM22, SM-MHC, smoothelin, and calponin): laminar flow reduces expression of all five SMC marker genes in SMCs and MFBs on a 2-D substrate; interstitial flow attenuates gene expression of SM-MHC, smoothelin, and calponin, but enhances expression for α-SMA and SM22. The influences of laminar flow and interstitial flow on expression of SMC marker genes are mediated by activation of ERK1/2 MAPK. In addition, cell surface glycocalyx HSPGs play a major role in mechanotransduction of fluid flow-induced ERK1/2 activation. SMCs and MFBs have the same pattern of phenotypic modulation in response to fluid flow.

Vascular SMC dedifferentiation, migration, proliferation, and protein secretion play central roles in both vascular remodeling and vascular lesion formation [1]. Phenotypic modulation (switching) is one of the key events for SMCs to be engaged in vascular repair, remodeling, and disease. In response to vascular injury, contractile SMCs are capable of transiently modulating their phenotype to a highly synthetic state with increasing ability to migrate into wound sites. In vivo, SMCs continuously encounter mechanical stimuli that play important roles in governing cell function and phenotype [3]. Surgical intervention such as balloon angioplasty or stent implantation can denude endothelial cells and damage the intima, leaving SMCs directly exposed to luminal blood flow shear stress. In hypertension, SMCs and FBs/MFBs are not only exposed to tensile stress (stretch), but also exposed to elevated interstitial flow driven by augmented transmural pressure [5], [19]. Therefore, in the early stages of vascular injury, shear stress induced either by luminal blood flow or by transmural flow may alter the phenotype of SMCs and FBs/MFBs. In the present in vitro study, we show that both laminar flow (2-D) and interstitial flow (3-D) affect expression of SMC marker genes in SMCs and MFBs. Downregulation of the contractile marker genes induced by fluid flow shear stresses indicates that fluid flow promotes SMC/MFB phenotype switching from a contractile state to a more synthetic and proliferative state. This suggests that in the early stage of vascular injury, fluid flow shear stress plays a role in vascular SMC and MFB phenotypic modulation and therefore contributes to vascular repair or vascular lesion formation.

Other studies have shown that FBs can differentiate into MFBs followed by further differentiation into SMC like cells [7]. Vascular SMCs, FBs, and MFBs therefore share common characteristics and functions. α-SMA is widely expressed in both SMCs and MFBs [13] and regulates cell contractility when it is incorporated within actin filaments to form stress fibers. SM22 is highly expressed in SMCs, FBs, and MFBs [32]. SM22 colocalizes with α-SMA and may play a role in actin filament remodeling, but it is not essential for SM development and its function still remains unknown [33]. Calponin is also expressed in SMCs and FBs/MFBs [34]. SM-MHC and smoothelin are better SMC markers, and smoothelin protein is thought to be only expressed in mature and fully differentiated SMCs [35]. However, it has been shown that FBs/MFBs also express SM-MHC and smoothelin [36]. In the present study we also detected the expression of these genes in MFBs by RT-qPCR.

Laminar flow reduces expression of all studied SMC marker genes, consistent with several studies [15], [16]. Other 2-D studies, however, have shown that shear stress can reduce cell proliferation [37], [38] and induce apoptosis [39]. The controversy about different effects of shear stress on SMC proliferation probably is due to the level of shear stress and the patterns of shear stress that were applied to cells, and also the species and phenotypic states of SMCs that were used. In this study, 3-D interstitial flow attenuates expression of SM-MHC, smoothelin, and calponin genes, but enhances expression for α-SMA and SM22. The disparity between 2-D and 3-D suggests the microenvironmental cues that cells receive are important for phenotype modulation. In conventional 2-D cultures, decreased cell proliferation is generally associated with the contractile state. In 3-D, SMC proliferation and cytokine secretion are reduced, suggesting that cells are more contractile compared with 2-D. However, α-SMA expression is also reduced in 3-D [3], [9]. This seeming contradiction may well explain the differences in gene expression between cells in 2-D and 3-D in response to flow. SMCs cultured in a 3-D collagen matrix express diminished α-SMA, and exhibit less phosphorylation of focal adhesion kinase [9], [40] and less spreading. It has been suggested that incorporation of α-SMA into stress fibers directly correlates with the strength of cell-matrix adhesion and is crucial for cell contractility and cell spreading [41]. In our study, when exposed to interstitial flow, cell proliferation and spreading are increased (data not shown), and therefore more cell-matrix adhesions are required, which could induce α-SMA and SM22 expression to promote stress fiber formation and cell-matrix adhesion formation. This is consistent with another study that has shown that interstitial flow can increase α-SMA expression and promote dermal FBs differentiation into MFBs and enhance their proliferation in 3-D collagen gels [42].

It has been suggested that laminar flow shear stress can induce SMC to decrease expression of SMC markers and increase expression of endothelial cell (EC) markers which transdifferentiate SMCs into ECs [16]. Because a 3-D environment is suitable for SMC and MFB physiology and a 2-D system is more suitable for ECs to perform their function, another explanation for our observations is that reduction in SMC marker expression on 2-D substrates turns SMCs/MFBs into more EC like cells; while interstitial flow only modulates SMC/MFB in 3-D from a more contractile phenotype to a more synthetic state.

SM22 and α-SMA are not SMC specific markers and are present in many cell types. Their roles generally are related to stress fiber formation and cell contractility, which may regulate cell motility. This hypothesis may well explain the difference in flow-induced migration between 2-D and 3-D that we have observed before: in 2-D studies, laminar flow-induced downregulation of α-SMA and SM22 may inhibit SMC and MFB migration [13], [18], while in 3-D, interstitial flow-induced upregulation of α-SMA and SM22 may enhance SMC and MFB motility [5].

In 3-D, unlike SM-MHC, smoothelin and calponin, α-SMA and SM22 were upregulated by interstitial flow and that could not be reversed by PD98059 or heparinase; instead, their expression was further enhanced (Figure 4). However, the interstitial flow-upregulated expression could be abolished by specific knockdown of ERK1/2 (Figure 5), indicating that besides affecting ERK1/2, PD98059 and removal of HSPGs by heparinase also affected other cellular signaling pathways which induced expression of α-SMA and SM22. We observed that treatment with PD98059 and heparinase caused cells to contract slightly in 3-D gels (data not shown). In 3-D, cell-matrix adhesions are smaller and there is less phosphorylation of focal adhesion kinase at tyrosine 397 than in 2-D [40], [43], suggesting the strength of attachments is weaker in 3-D than in 2-D. In 3-D, cells treated with PD98059 contracted slightly due to toxicity. Cleavage of HS GAGs by heparinase reduced cell attachments to ECM through HSPGs (such as syndecans) [44]. Also note that proliferation of SMC/MFB in 3-D was reduced compared with 2-D [9] and that cell physiology is quite different in 2-D and 3-D [43], [45]. Treatment with PD98059 and heparinase further reduced cell proliferation and decreased cell attachment to ECM in 3-D. When exposed to interstitial flow, cells tended to be more proliferative and spread out to regain attachments to ECM, which likely required an increase in expression of α-SMA and SM22 triggered by some signaling pathways other than ERK1/2 for stress fiber and cell-matrix adhesion assembly. The exact mechanism by which PD98059 and heparinase affect cells differently in 3-D remains unclear. However, since ERK1/2 knockdown is more specific to ERK1/2 and is able to reverse flow effects on gene expression in both 2-D and 3-D, this suggests that flow modulation of SMC marker expression is dependent on ERK1/2 in both 2-D and 3-D.

MAPKs are important mediators which regulate a variety of cellular processes, including gene expression, proliferation, survival, apoptosis, migration, and differentiation [46]. In cardiovascular health and disease, it has been shown that the ERK1/2 MAPK pathway plays an important role [47]. Increased ERK1/2 activation contributed to augmented vascular SMC proliferation and neointima formation with aging in a rabbit study [48]. The rapid activation of ERK1/2 after balloon injury of the rat carotid artery may be associated with vascular SMC migration and proliferation in vivo [49]–[51]. The evidence from in vitro studies has also shown that ERK1/2 activation plays a crucial role in cardiovascular cell proliferation and cell migration [15], [19]. The increase in SMC proliferation and migration is associated with a phenotypic switching from the contractile state to the synthetic state, which is regulated by sustained phosphorylation of ERK1/2 [52]. Shear stress (∼10 dyn/cm^2^) can induce early growth response-1 expression in SMCs via activation of ERK1/2 and c-Jun within 1 h [53] and promote SMC proliferation wthin 1 day [15]. Our results suggest that besides the role in regulating cell proliferation as reported in the literature, ERK1/2 also plays a central role in both laminar flow and interstitial flow-induced cell phenotype modulation.

In 2-D studies, it has been suggested that the cell surface glycocalyx is responsible for sensing fluid flow shear stress on vascular ECs and SMCs [20]–[22]. In 3-D, HSPGs, which are present all over the cell surface, bind extracellular ligands and form signaling complexes with receptors. Binding of cell surface HSPGs to ECM components can immobilize the proteoglycans, enabling HSPG core proteins to interact with the actin cytoskeleton [54]. Therefore, the cell surface HSPGs can act as both coreceptors and mechanosensors in ECM-cytoskeleton interactions. In the present study, we demonstrated that cell surface HSPGs are flow sensors by which cells can sense laminar flow and interstitial flow stimuli and then undergo phenotypic modulation through ERK1/2 activation.

We have shown previously that 1–6 h of interstitial flow driven by 1 cmH_2_O pressure differential (∼0.05 dyn/cm^2^) can dramatically enhance cell motility and MMP expression [5]. In the present study, we again show that this short exposure to interstitial flow also has great impact on the expression of cell phenotypic genes. The unique 3-D cell-matrix structure may play the key role. In the more physiological 3-D system, the cells exhibit matrix adhesions all over their surface, and 3-D cell-matrix interactions enhance cell biological activity [40]. Thus the mechanosignal of interstitial flow on the glycocalyx can be significantly amplified by interactions between the cell cytoskeleton and matrix through cell-matrix adhesions.

In summary, we have shown, for the first time, that both laminar flow and interstitial flow are capable of modulating SMC and MFB phenotype into a more synthetic state via HSPG-mediated ERK1/2 activation - a mechanotransduction mechanism. Fluid flow-induced phenotype modulations are somewhat different in 2-D and 3-D: fluid flow down-regulates both α-SMA and SM22 in 2-D, but promotes their expression for cell spreading in 3-D; however, fluid flow reduces expression of more specific SMC markers (such as SM-MHC and smoothelin) in both 2-D and 3-D. On the other hand, interstitial flow can induce FB differentiation into MFB in 3-D [42]. Together with the fact that laminar flow inhibits SMC and MFB migration in 2-D [13], [18] and interstitial flow can enhance SMC, FB, and MFB motility in 3-D [5], [19], our study may indicate that during vascular injury, in response to the alterations of interstitial flow in the local environment, SMCs in the media can shift their phenotype from a contractile state to a more synthetic state and FBs in the adventitia can modulate their phenotype from a quiescent state to an activated state and differentiate into MFBs. Under the sustained stimulation of interstitial flow, the synthetic SMCs and activated FB and MFB gain higher motility and migrate into the intima or wound sites. While for the superficial layer of SMCs in the injury regions, the luminal blood flow directly promotes their dedifferentiation into a more proliferative state and inhibits their migration. SMCs and MFBs in the intima or injury sites can proliferate, secrete ECM proteins, and increase stress fiber contractility by expressing α-SMA under interstitial flow, which therefore contribute to wound closure and healing, vascular remodeling, or vascular lesion formation. This study also suggests that ERK1/2 and cell surface HSPGs may be the potential targets for regulation of cell phenotype and inhibition of vascular lesion formation.

This is the first study to describe a flow-induced mechanotransduction mechanism regulating vascular SMC and MFB differentiation in both 2-D and 3-D. HSPGs present on the surfaces of many types of cells (such as epithelial cells, cardiovascular cells, tumor cells, and stem cells) and play important roles in cell growth, adhesion and migration, regulating development, tumorigenesis, and vasculogenesis [44], [54], [55]. Therefore, our study will be of interest in understanding the flow-related and HSPG-regulated mechanotransduction mechanisms in vascular lesion formation, tumor cell invasion, and stem cell differentiation.

## Materials and Methods

### 2-D and 3-D cell culture

Rat aortic SMCs and MFBs were obtained, characterized, and cultured as previously described [13]. For 2-D experiments: SMCs and MFBs were seeded on fibronectin coated (30 µg/insert) 6-well format cell culture inserts with 0.4 µm pore size (1.5×10^5^ cells/insert) and cultured for 24 h with 2 ml of growth medium in the inserts and 3 ml of growth medium in the companion well. For 3-D experiments: SMCs and MFBs were suspended in rat tail collagen I (BD Science) gels and plated in 6-well cell culture inserts with 8 µm pore size (cell density: 2.5×10^5^ cells/ml; gel volume: 1 ml; final gel concentration: 4 mg/ml); cells were then cultured for 24 h with 2 ml growth medium in the bottom well [19].

### Fluid flow shear stress experiment

2-D laminar flow: a rotating disk shear rod device was used [18], and the average shear stress of 8 dyn/cm^2^ was applied to cells cultured in the inserts for 15 h. 3-D interstitial flow: cells in 3-D collagen gels were subjected to interstitial flow as previously described [5] for 6 h, which was driven by a 1 cmH_2_O pressure differential (∼0.05 dyn/cm^2^). There have been no experimental measurements of shear stress on SMCs and MFBs, therefore the exact shear stresses are not known. However, the levels of shear stress we used in this study have been used in many other studies and are within modeling predictions and thought to be physiological [5], [10], [15], [19], [39], [53].

### ERK1/2 inhibition and HSPG cleavage

PD98059 (Calbiochem) was used for ERK1/2 inhibition and heparinase III (IBEX Technologies, Montreal, Canada) was used for HSPG cleavage. After 24 h spreading in 2-D or 3-D, cells were pre-incubated with 10 µM of PD98059 or 6.7 IU/L of heparinase III for 3 h in growth medium. Cells were then subjected to flow experiments as described above.

### RNA interference

To silence ERK1/2, two ERK1 short hairpin (sh) RNAs and two ERK2 shRNAs which were subcloned into pSUPER vector (kindly donated by Dr. Michal Hetman) and were co-transfected into SMCs. The ERK1/2 shRNA target sequences were:

shERK1-1, GACCGGATGTTAACCTTTA;

shERK1-2, ATGTCATAGGCATCCGAGA;

shERK2-1, GTACAGAGCTCCAGAAATT;

and shERK2-2, AGTTCGAGTTGCTATCAAG [19], [56].

The transfections were conducted using Lipofectamine™ LTX and PLUS™ reagents (Invitrogen).

### RNA extraction and gene expression analysis

2-D experiments: 0.5 ml of TRIzol® LS Reagent (Invitrogen) was added to cell culture inserts and incubated for 5 min with gentle pipette mixing; samples were then transferred to microcentrifuge tubes for further RNA extraction. 3-D experiments: Cells in collagen gels were directly lysed by TRIzol and the insoluble materials were removed by centrifugation at 12,000×g for 10 minutes at 4°C. Chloroform was added for phase separation followed by RNA isolation using the Purelink RNA Mini Kit (Invitrogen). RNA samples were then converted to cDNA by reverse transcription (RT). For analyzing gene expression, quantitative polymerase chain reaction (qPCR) was performed using the following protocol as previously described [19]: GAPDH served as an internal control; reactions were performed in 25 µl reaction mixture volumes containing ABsolute Blue QPCR SYBR Green ROX Mix (Thermo Scientific), cDNA and specific primer pairs; real-time PCR protocol was set to 15 minutes at 95°C followed by 45 cycles of 30 seconds at 95°C, 30 seconds at 55°C, and 30 seconds at 72°C; the fluorescent data were collected at 80°C; the dissociation curve analysis was used to assess the specificity of product amplification. Primer sequences are listed in Table 1.

**Table 1:** Primer sequences for rat SMC marker genes.

Gene	Forward sequence (5′-3′)	Reverse sequence (5′-3′)	GenBank Locus	Reference
α-SMA	GATCACCATCGGGAATGAACGC	CTTAGAAGCATTTGCGGTGGAC	NM_031004.2	[57]
SM22	TGTTCCAGACTGTTGACCTC	GTGATACCTCAAAGCTGTCC	NM_031549.2	[58]
SM-MHC	AAGCAGCTCAAGAGGCAG	AAGGAACAAATGAAGCCTCGTT	NM_001170600.1	[59]
SMTN	TCGGAGTGCTGGTGAATAC	CCCTGTTTCTCTTCCTCTGG	NM_001013049.2	[60]
Calponin	ACAAAAGGAAACAAAGTCAAT	GGGCAGCCCATACACCGTCAT	NM_031747.1	[61]
GAPDH	TCTTCACCACCATGGAGAA	ACTGTGGTCATGAGCCCTT	NM_017008	[5]

### Immunofluorescence staining

To assess the cleavage of heparan sulfate glycosaminoglycans (HS-GAG) by heparinase III, primary antibody HepSS-1 (US Biological) and secondary antibody Alexa Fluor 350 goat anti-mouse IgM (Invitrogen) were used to stain HS-GAGs. Briefly, SMCs were seeded in plate wells for 2 days and then treated with 6.7 IU/L heparinase III for 1 hour, followed by fixation with 4% paraformaldehyde and blocking with 4% BSA in PBS. Then cells were incubated with primary antibody HepSS-1 (1∶200 dilution in PBS with 4% BSA) for 2 hours and secondary antibody anti-mouse IgM (1∶100 dilution) for 2 hours at room temperature. Finally, cells were mounted with mounting medium containing propidium iodide (PI) (Vector Laboratories) and covered by coverslips.

### Protein extraction and Western blotting

Protein extraction from 2-D: briefly, after washing cells in inserts with ice-cold PBS, 1× lysis buffer was added and cell scrapers were used to remove cells from inserts; samples were sonicated for 30 s and rocked for 15 min; supernatants were collected and cell pellets were discarded by centrifugation. Protein extraction from 3-D collagen gels was described previously in detail [19]: briefly, 2× lysis buffer (with a supplement of 2× protease inhibitor cocktail and 2× phosphatase inhibitor cocktail, 2 mM activated Na_3_VO_4_, and 2 mM PMSF) was added immediately to the gels followed by sonication for 45 s on ice; lysates were centrifuged at 12,000 g for 1 hour at 4°C, and then the supernatants were collected and the remaining gel pellets were discarded; the supernatants were concentrated using Centrifugal Filter Units (Millipore); the protein samples were boiled for 5 minutes after mixing with 4× sample buffer and then subjected to SDS-PAGE; proteins were transferred to PVDF membranes and incubated with specific primary antibodies (ERK1/2 and phospho-ERK1/2, from cell signaling), followed by incubation with an ECL horseradish peroxidase (HRP)-linked anti-rabbit IgG antibody (Amersham, GE Healthcare); the proteins on PVDF membranes were then detected using Immobilon Western Chemiluminescent HRP Substrate (Millipore) and the ChemiDoc XRS system with the Quantity One software (Bio-Rad); some membranes were stripped using Restore™ Plus Western Blot Stripping Buffer (Thermo Scientific Pierce) for a subsequent detection.

### Data Analysis

Results are presented as mean ± SEM. Data sets were analyzed for statistical significance using a Student's t-test with a two-tailed distribution, and *P*<0.05 was considered statistically significant.
